# Zirconia–Alumina Composites Obtained by Centrifugal Slip Casting as Attractive Sustainable Material for Application in Construction

**DOI:** 10.3390/ma14020250

**Published:** 2021-01-06

**Authors:** Justyna Zygmuntowicz, Justyna Tomaszewska, Radosław Żurowski, Marcin Wachowski, Paulina Piotrkiewicz, Katarzyna Konopka

**Affiliations:** 1 Faculty of Materials Science and Engineering, Warsaw University of Technology, 141 Woloska Str., 02-507 Warsaw, Poland; paulina.piotrkiewicz.dokt@pw.edu.pl (P.P.); Katarzyna.Konopka@pw.edu.pl (K.K.); 2 Instytut Techniki Budowlanej, Ksawerów 21, 02-656 Warsaw, Poland; j.tomaszewska@itb.pl; 3 Faculty of Chemistry, Warsaw University of Technology, 3 Noakowskiego Str., 00-664 Warsaw, Poland; rzurowski@ch.pw.edu.pl; 4 Faculty of Mechanical Engineering, Military University of Technology, 2 Gen. S. Kaliskiego Str., 00-908 Warsaw, Poland; marcin.wachowski@wat.edu.pl

**Keywords:** zirconia–alumina composites, centrifugal slip casting, life cycle assessment (LCA)

## Abstract

This paper focuses on the possibility of adapting the centrifugal slip casting method to obtain zirconia–alumina composite materials in the form of finished tube-shaped products. These types of products, due to their unique properties, can be utilised, for example, in the transport of aggressive substances, even in extreme temperatures or corrosive conditions. The study reports on the two series of zirconia–alumina composites differing in the content of ZrO_2_—2.5 and 25 vol%. The fabricated and sintered materials were characterised using scanning electron microscopy (SEM), X-ray diffraction (XRD) and stereological analysis. Moreover, a life cycle assessment (LCA) was provided in accordance with the requirements of the ISO 14044 and EN 15805 standards. The obtained data clearly show that the centrifugal slip casting method allows obtaining samples with high density and extremely uniform distribution of the ZrO_2_ phase in the alumina matrix. The stereological analysis results proved also that the addition of ZrO_2_ is effective in reducing the growth of Al_2_O_3_ grains during the sintering process. The phase analysis carried out by means of XRD showed that during the sintering process, in the case of composites with a lower ZrO_2_ content (2.5 vol%), the monoclinic to tetragonal transformation of ZrO_2_ was total, while for samples containing 25 vol% ZrO_2_, the monoclinic phase remained in a small amount in the final product.

## 1. Introduction

Research on modern composite materials is currently one of the fastest growing fields of material engineering. Technological developments in the field of materials manufacturing make the search for unconventional solutions in this area essential. The basic direction of the development of modern technologies is based primarily on the search for new, advanced materials. Composites owe their dynamic development mainly to their unique properties, that is, high hardness and mechanical strength, chemical and thermal resistance, and resistance to abrasive wear and corrosion at elevated temperatures. Functional ceramic–ceramic composites [1] have the potential to provide a microstructure that is optimally suited to these requirements. Research is constantly being conducted to increase our knowledge on the correlation between certain parameters of the technological process, with the aim of achieving a microstructure with the desired properties. The right correlation of these parameters facilitates the controlled and planned development of a structure that meets the needs and desired properties of the material. It should be remembered, however, that these correlations are closely related to the type of material forming method applied. One of the fundamental factors in choosing the method of producing functional composite materials is the economic aspect. The costs associated with the method of manufacturing such materials largely determine the material’s anticipated longevity. More importantly, the method being used should be able to produce a material with high repeatability and efficiency. Due to the ongoing transition of EU member states towards the circular economy (CE) model, the environmental impact associated with the manufacturing process also requires investigation [2,3,4]. The implementation of composite materials in the industry leads to a reduction in costs associated with the operation and maintenance of different machine components.

The growing demand of many industries for functional composite materials with customised, unique properties requires further knowledge in this area. In-depth understanding of the relationship between the selection of raw materials, the choice of the moulding and sintering methods and the parameters for conducting these processes, as well as the final properties of the products obtained, is crucial in maximising their potential. For this reason, functional composite materials are still the subject of ground-breaking research. 

According to the current state of knowledge, ceramic–ceramic composite materials can be formed by a variety of methods. The most frequently used methods include hot pressing [5,6], extrusion [7,8], injection moulding [9,10], slip casting [11,12], tape casting [13,14], electrophoretic deposition [15,16] or centrifugal slip casting [17,18].

The results of our own research on the development and application of the centrifugal slip casting method for the production of composites will allow us to obtain a finished product in the shape of a sleeve. The main aspect in favour of applying this technology to the manufacture of composites is the ability to form a shape in a single operation. The developed technology enables the formation of pipes, where the particles, under the influence of the centrifugal force, are packed into free space under reduced coefficients of friction, which effectively minimises the residual stress in the piece [19,20]. Residual stress has a negative impact on the microstructure and mechanical properties of the moulded pieces, as their relaxation causes deformation and cracking. As a result, a rigid composite casting is obtained, which is characterised by high mechanical strength and the presence of a metallic phase gradient in the material. Until now, this method has only been used to produce composites from the ceramic–metal system. In this article, the research will focus on extending the experiments conducted so far to new material systems such as ceramic–ceramic composites. It is worth noting that there are no publications concerning the production of this type of material by centrifugal casting.

The present article deals with the adaptation of the existing centrifugal slip casting method to suit a new system, namely, Al_2_O_3_–ZrO_2_ (i.e., zirconia–alumina composites).

Corundum ceramics are currently the most common and most frequently used ceramic construction material due to their low price and availability [1,7,11]. Al_2_O_3_ is a material whose manufacturing technology has been mastered on a large scale [7,11]. Due to their mechanical properties and corrosion resistance, ZrO_2_ ceramics and their composites have become attractive constructional and functional materials with a reputable standing in the industry. Materials based on ZrO_2_ are used in furnace and motor components, in abrasion-resistant elements or in cutting tool blades [21,22,23,24,25]. It is worth noting that ZrO_2_ comes in three polymorphic varieties [26]. The monoclinic structure is available in the low-temperature range. As the moulding temperature increases, a variety with tetragonal symmetry appears. In turn, in the highest temperature range up to the melting point of 2988 K, a permanent variety with a regular fluorite structure occurs. During the transition from the tetragonal to the monoclinic phase, microcracks may appear as a result of the generation of stress that correlates with an increase in cell volume from 3% to 5% [26]. Literature data indicate that the presence of certain oxides at high temperature in the range of 1273 K to 1773 K causes the stabilisation of the regular and tetragonal varieties of ZrO_2_ [26,27]. The transformation between polymorphic varieties occurs under stress (such as martensitic transformation) and it is a very effective mechanism for increasing resistance to cracking in composites.

The aim of this research is to use the centrifugal slip casting method to produce zirconia–alumina composites with different proportions of Al_2_O_3_ and ZrO_2_ phases. Moreover, an analysis of the influence of solid phase content on the microstructure and selected properties of zirconia–alumina composites formed by centrifugal slip casting will be performed. The conducted research will help us learn about the correlation between the phase composition, microstructure and basic properties of the manufactured composites. Furthermore, the environmental impact accompanying the composites’ manufacturing process has been determined using the life cycle assessment (LCA) method, in accordance with the ISO 14044 and EN 15804 standards.

## 2. Materials and Methods

The following commercially available powders were used to produce the composites: TM-DAR aluminium oxide by Taimei Chemicals Co., Tokyo, Japan, with an average particle size of 120 ± 30 nm, and zirconium oxide by Tosoh Co., Tokyo, Japan, stabilised by 3% mol Y_2_O_3_ with an average particle size of about 40 nm, symbol TZ-PX-245. The Al_2_O_3_ and ZrO_2_ powders used have a purity of 99.99%. According to the manufacturer, the ZrO_2_ tetragonal phase containing 3% yttrium oxide as a stabiliser has an actual density of approximately 6.05–6.10 g/cm^3^, while α-Al_2_O_3_ has a density of 3.98 g/cm^3^. Yttrium oxide introduced into zirconium dioxide guarantees its complete stabilisation and prevents any polymorphic changes of ZrO_2_ while cooling to room temperature [26,27]. The choice of the Al_2_O_3_ TM-DAR and ZrO_2_ TZ-PX-245 powders was mainly dictated by their very high purity and nanometric (ZrO_2_) and submicrometric (Al_2_O_3_) particle size. In addition, these powders are often used by scientists, thus allowing for subsequent comparisons to be made with international research results.

In preparing the composites, 50 vol% solid phase aqueous casting slips were prepared containing a mixture of ceramic powders. Two sample series were prepared which differed in zirconium oxide volume content: Series I contained 2.5 vol% ZrO_2_ and Series II contained 25 vol% ZrO_2_ in relation to the total solid phase content. Diammonium citrate (DAC) from PoCH and citric acid (CA) from Sigma-Aldrich (St. Louis, MO, USA) were used as liquefiers. The amount of liquefiers was chosen experimentally and determined at 0.3 wt.% DAC and 0.2 wt.% CA in relation to the solid phase. A 10% aqueous solution of poly(vinyl alcohol) (PVA) was used as a binder and added to the slip in the amount of 3 wt.% in relation to the solid phase. Deionised water was used as solvent.

The first step in the preparation of ceramic casting slips involved filling a sintered corundum container with deionised water, liquefiers, the binder, and composite powder, which was added in batches. The casting slip thus prepared was mixed in a Retsch PM 400 planetary ball mill (Germany) at a speed of 300 rpm for 1 h. Then, in order to remove air bubbles from the suspension, the slip was deaerated in a Thinky Corporation (Tokyo, Japan) THINKY ARE-250 device. Deaeration of the slip was carried out for 11 min at 2200 rpm. The THINKY ARE-250 allows releasing bubbles > 1 μm from the suspensions, whereby it reduces the probability of defects in the zirconia–alumina materials. In the next stage of composite formation, the prepared homogeneous slip was poured into a gypsum mould placed in the metal casing of the centrifuge and subjected to centrifugal casting in a device specially prepared for this purpose. The process of centrifugal slip casting was carried out at a speed of 3000 rpm for 130 min at a temperature of 25 °C. Afterwards, the sample was removed from the device together with the gypsum mould and subjected to drying for 48 h at 30 °C. In the next step, the castings were removed from the mould and sintered. The sintering process of the composite castings obtained by centrifugal slip casting was carried out in a Carbolite S33 6RB furnace (Carbolite Gero Ltd., Hope, UK). They were heated to 1400 °C/min at a rate of 2 °C/min, then held at this temperature for 2 h, and finally cooled down at a rate of 5 °C/min. The sintering process was carried out in an atmosphere consisting of air.

Investigations first involved the characterisation of the ceramic powders. For this purpose, microscopic observations were made and the phase density and composition were determined. Microscopic observations of the powders were performed using a JSM-6610 (SEM JEOL) scanning electron microscope. The observations were conducted in secondary electron mode at 15 kV. The density of the initial powders was measured using an AccuPyc 1340 II helium pycnometer by Micrometrics (Norcross, GA, USA). The measurements were carried out in a helium environment, which was introduced into the measuring vessel under a pressure of 0.134 MPa. The measurement extended over a series of 700 cycles. Phase analysis of the powders was carried out by means of the X-ray diffraction method. The measurement was performed using a Rigaku Mini Flex II diffractometer (JEOL Ltd., Tokyo, Japan). The radiation source was an X-ray lamp with a copper anode and Kα radiation wavelength of λ = 1.54178 Å. The test was carried out in the θ 20–100° angular range at 30 kV potential and 15 mA current. The goniometer’s rotational speed was 0.02°/min and the diffraction signal countdown time was 3 s.

The obtained composite castings in the raw state were characterised using a macroscopic examination. Then, after sintering, linear shrinkage, volumetric shrinkage and density were determined. The latter was established by means of the pycnometric method. X-ray phase analysis (X-ray diffraction—XRD) was carried out to determine the phase composition and phase transformations occurring in the composites during sintering. The obtained castings were observed under a JEOL JSM-6610 scanning electron microscope (JEOL Ltd., Tokyo, Japan). Investigations were carried out with parameters analogous to those of the initial materials. During the observation, a voltage of 15 kV was used. The samples to be observed were carefully prepared. The sintered pieces in the form of pipes were cut into 1 cm tall fragments and were subsequently clamped. Fragments for metallographic examination were taken from the central part of the sample. The samples were then immersed in resin and ground on a Buehler grinding machine (Buehler, Esslingen, DE) using diamond suspensions of different grain sizes. A polishing slurry was added to the rotating disc and the samples were ground at an appropriate pressure for a specified period of time.

Stereological analysis was used to determine the average particle size of the composites and the effect of ZrO_2_ content on Al_2_O_3_ grain growth during the sintering process. Stereological parameters were determined using the MicroMeter v.086b [28,29] software based on fracture micrographs obtained with the SEM JEOL JSM-6610 microscope (JEOL Ltd., Tokyo, Japan). Observations were made of fractures sprayed with carbon. During image analysis, certain transformations were utilised allowing for complex operations necessary to determine the particle size. These included image transformations covering colour and contrast, opening, closing, dilation and erosion [28,29]. Such an analysis made it possible to obtain the grain size distribution of Al_2_O_3_ and ZrO_2_ in the composites.

The Vickers hardness was measured with the use of a Vickers hardness tester HPO-250 under the load of 196 N. The holding time of the load was 10 s. In the investigation for each sample, 15 hardness measurements were made. The results represented the average value of the hardness based on 15 indentation measurements.

Life cycle assessment (LCA) of the manufactured composites has been performed according to the requirements of ISO 14044 [30] and EN 15805 [31] standards and covers module 1—raw materials supply—and module A3—manufacturing. The impacts refer to the declared units (DU) of 1 sintered Al_2_O_3_-ZrO_2_ tube. The allocation rules used are measured on a mass basis. All impacts from raw materials extraction are allocated in module A1. Impacts associated with the laboratory-scale manufacturing process of Al_2_O_3_-ZrO_2_ composites are allocated in module A3. The packaging of raw materials was not taken into consideration. Life cycle inventory (LCI) data and emissions factors used for calculations come from Ecoinvent v.3.7, specific environmental product declarations (EPDs) [32] and the KOBiZE report 2019 [33].

## 3. Results and Discussion

**Figure 1:**
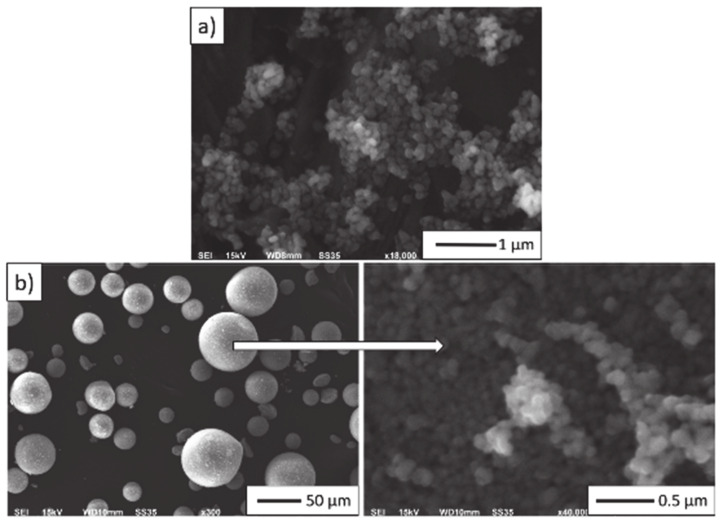
SEM micrographs: (**a**) aluminium oxide TM-DAR, (**b**) zirconium oxide TZ-PX-245.

The morphology of the powders visualised by means of the scanning electron microscope is shown in Figure 1. It is observed that both Al_2_O_3_ and ZrO_2_ powders tend to form agglomerates. In the case of alumina, its grain size was determined as ranging from 100 nm to 200 nm (Figure 1a). Microscopic observations revealed that zirconium oxide is a powder in granulate form consisting of nanometre-sized particles. Figure 1b revealed that ZrO_2_ powder particles form >30 μm granules consisting of fine powder in the size range of 50–100 nm. Micrographs have shown that the particle size of both powders corresponds to the size given by their manufacturer. The observations revealed that the particle shape of both powders is regular.

**Figure 2:**
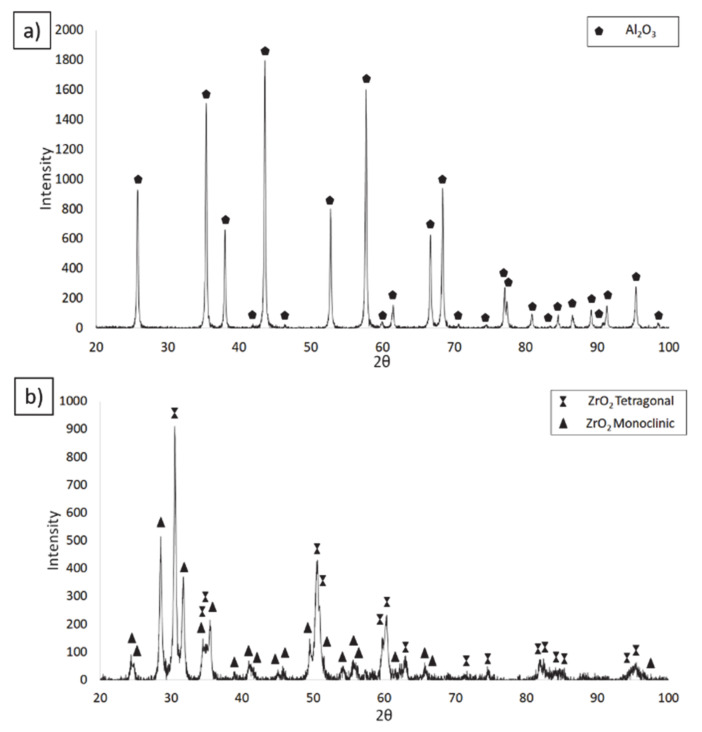
Phase composition analysis of powders (**a**) Al_2_O_3_, (**b**) ZrO_2_.

On the basis of pycnometric density results, it was found that Al_2_O_3_ had a density of 3.9701 g/cm^3^, while the value for ZrO_2_ was 5.8902 g/cm^3^. The obtained densities were slightly lower than those stated by the manufacturer. The differences between the density determined by the pycnometric method and the density stated by the manufacturer may result from insufficient desorption of the powder prior to measurement. This is particularly relevant for nanometric powders, which show significant surface development. If it is not possible to carry out a full desorption, the volume is inflated and, therefore, the density indicated by the device is lower. In addition, for the ZrO_2_ powder, the lower density value may be due to the powder’s phase composition. In order to check the phase composition of the tested powders, an XRD analysis has been carried out. The obtained results of the XRD analysis are shown in Figure 2. The performed tests demonstrated that ZrO_2_ powder contains two phases: a tetragonal phase (t-ZrO_2_, technical data sheet, PDF # 00-050-1089) and a monoclinic phase (m-ZrO_2_ technical data sheet, PDF # 04-007-9268). XRD analysis revealed that the ZrO_2_ powder contained 45.1 wt.% of the tetragonal phase and 54.9 wt.% of the monoclinic phase (Figure 2b). This means that the zirconium oxide powder is not fully tetragonal and, therefore, the presence of a monoclinic phase reduces the average density of the ZrO_2_ powder mixture stated by the manufacturer.

**Figure 3:**
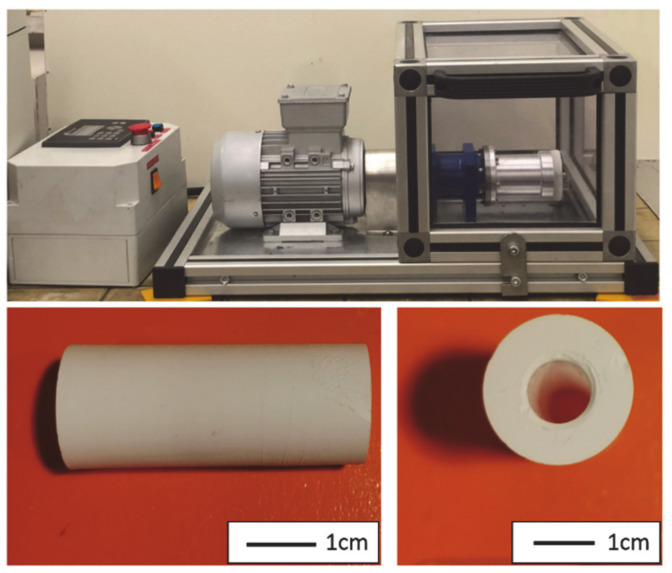
Device for manufacturing composites and finished Al_2_O_3_–ZrO_2_ (Series II) composite product in the shape of a sleeve made by centrifugal slip casting.

The adapted centrifugal slip casting method consists of powder consolidation by centrifugal force with densification of the product by capillary forces, which remove liquid from the suspension. The proposed technological process makes it possible, in the first stage, to obtain a stiff raw composite casting in the shape of a sleeve that accurately reproduces the shape of the mould. The raw piece is then subjected first to drying and then sintering. At the current stage of development and with the equipment available, this method makes it possible to produce composite elements in the shape of a sleeve with a length of about 4 cm and an external diameter of 2 cm. Figure 3 shows an example photograph of a sample (Series II—25 vol% ZrO_2_) post-sintering obtained via centrifugal slip casting. Based on a macroscopic examination, no visible cracks or defects were observed on the surface of the produced composite, which confirms the usefulness of the centrifugal slip casting method for producing a finished product in the shape of a sleeve (e.g., for transporting aggressive media at elevated temperatures).

Table 1 presents the results of density and shrinkage measurements for zirconia–alumina composites. On the basis of density measurements by pycnometer, it was found that the relative density increases with decreasing ZrO_2_ content in the sinter. The results obtained are in accordance with the classical sintering theory, according to which the dispersed phase grains interfere with the majority phase densification. It has been noted that Series I composites (2.5 vol% ZrO_2_) were characterised by an almost total density of over 99%, whereas in Series II (25 vol% ZrO_2_), a relative density of 98% was achieved. The density values obtained are the result of, among other things, the compaction of the particles by centrifugal force during moulding. Volumetric and linear shrinkage measurements of the sintered samples were also performed. For composites containing 25 vol% ZrO_2_, a volumetric shrinkage of 35.58% and a linear shrinkage of about 14.4% were obtained. Slightly lower volumetric shrinkage (34.19%) and linear shrinkage (13.1%) were obtained for samples containing 2.5 vol% ZrO_2_.

**Figure 4:**
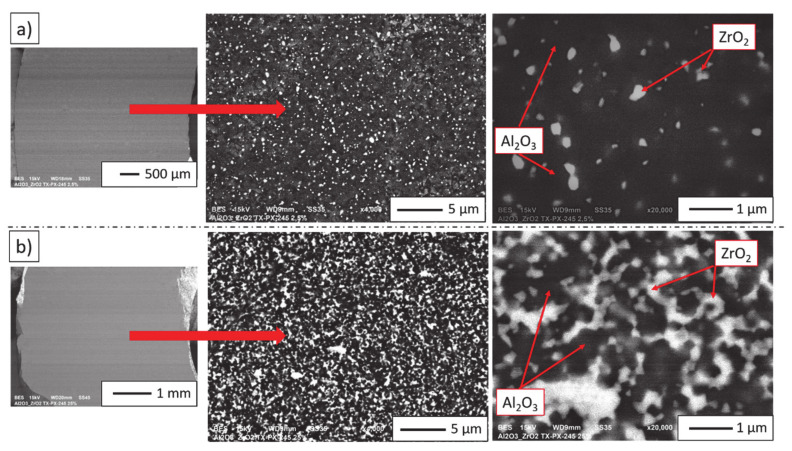
SEM micrograph of zirconia–alumina composites: (**a**) Series I—2.5 vol% ZrO_2_, (**b**) Series II—25 vol% ZrO_2_.

Figure 4 shows the selected images presenting the microstructure of ceramic castings formed by centrifugal slip casting of zirconia–alumina composites containing 2.5 vol% and 25 vol% ZrO_2_. Bright areas on the micrographs correspond to phase ZrO_2_, while grey areas to phase Al_2_O_3_. Based on the microscopic observations of the composites after sintering, it can be stated that all castings were characterised by a homogeneous microstructure. It was observed that regardless of ZrO_2_ content, the composites were characterised by a homogeneous distribution of ZrO_2_ in the Al_2_O_3_ matrix. Moreover, literature findings indicate that in zirconia–alumina composites shaped pieces obtained by pressing, ZrO_2_ grains are not as homogeneously dispersed in the Al_2_O_3_ matrix as they are in the composites obtained by centrifugal slip casting [34,35,36].

**Figure 5:**
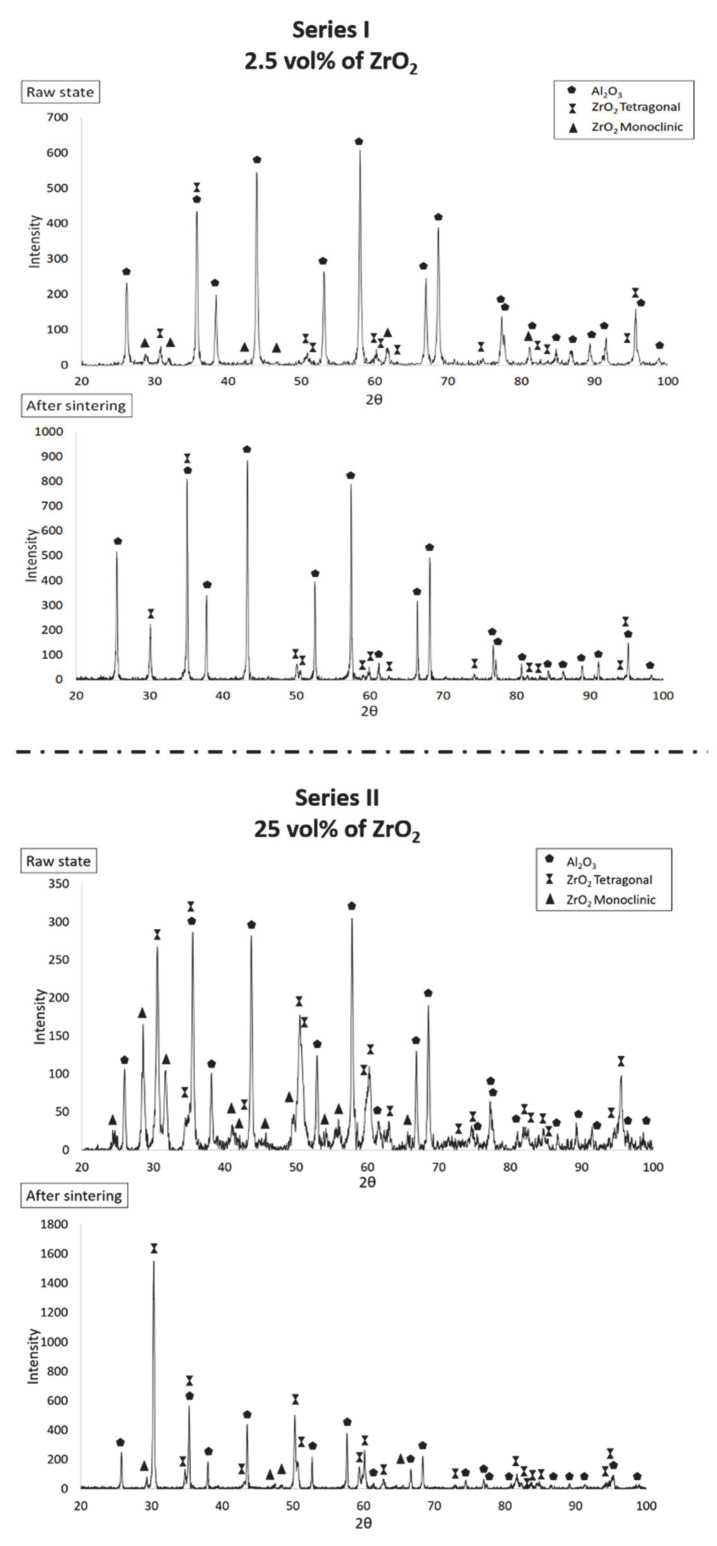
Summary of diffractograms for zirconia–alumina composites with different content of ZrO_2_ phase before and after sintering.

Figure 5 shows the diffractograms of the selected samples. It was found that the diffractograms of samples from both series before and after sintering showed significant differences. It was observed that in Series I with 2.5 vol% ZrO_2_, three phases were present before sintering: Al_2_O_3_, t-ZrO_2_ and m-ZrO_2_. After sintering Series I samples, only aluminium oxide and the tetragonal variety of zirconium oxide occurred. On the basis of literature findings, it is clear that depending on temperature, ZrO_2_ occurs in three polymorphic varieties: monoclinic, tetragonal and regular [37,38]. The monoclinic variety of ZrO_2_ is durable at low temperatures. Heating it to about 1200 °C leads to its transition to the tetragonal variety, which is beneficial in terms of mechanical properties. The tetragonal variety is stable up to about 2370 °C [37,38]. In addition, the transition from the tetragonal to the monoclinic phase is characterised by its nondiffusive nature and the fact that the start transformation temperature hysteresis appears at this point. This transformation is a reversible martensitic transformation [39,40,41,42,43]. In zirconia–alumina composites containing 2.5 vol% ZrO_2_, it was found that there was a complete transformation of the tetragonal phase into the monoclinic ZrO_2_ phase as a result of sintering at 1400 °C.

In Series II samples containing 25 vol% ZrO_2_, three phases were observed both before and after the sintering process: Al_2_O_3_, t-ZrO_2_ and m-ZrO_2_. The obtained diffractograms are similar and differ from the tetragonal and monoclinic phases only in reflex intensity. XRD analysis results showed that in the raw Series II samples, the t-ZrO_2_ phase content was 18.9 wt.%, while the content of m-ZrO_2_ was 15.3 wt.%. In Series II sample composites, after sintering, as a result of the transition from the tetragonal to the monoclinic phase, the content of t-ZrO_2_ was 30.7 wt.%, while the content of m-ZrO_2_ was 0.9 wt.%.

**Figure 6:**
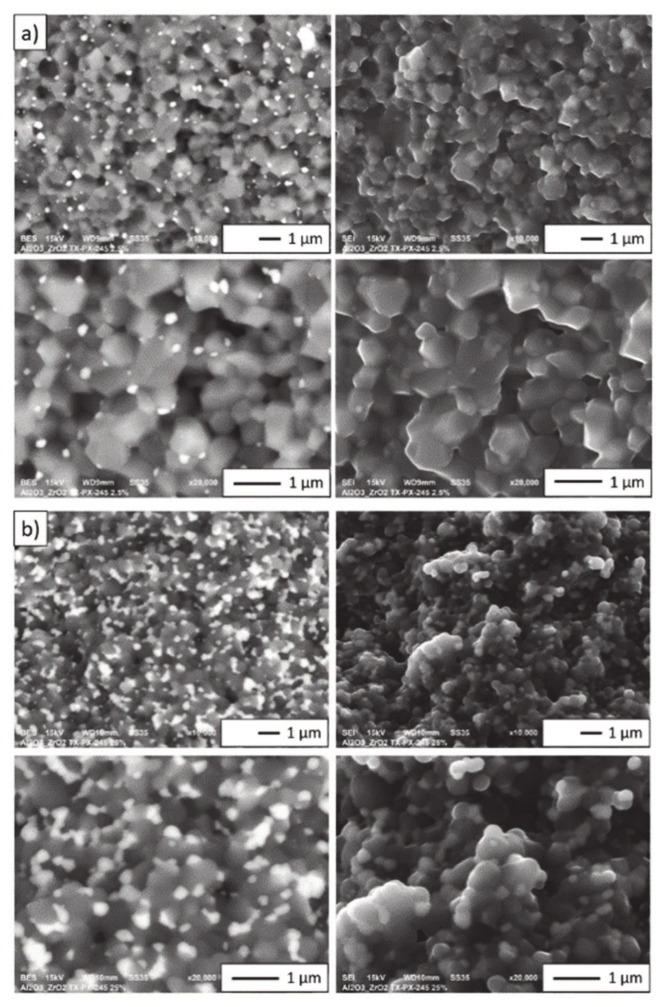
SEM images of: (**a**) Series I (2.5 vol% ZrO_2_), (**b**) Series II (25 vol% ZrO_2_), illustrating the differences in Al_2_O_3_, ZrO_2_ grain size.

Fracture microstructures of zirconia–alumina composites (Series I and II) are presented in Figure 6. Fractures in the studied composites are of an intergranular brittle nature. Intercrystalline fractures are visible along the grain boundaries. Moreover, the fracture surfaces are smooth and in the form of polyhedrons corresponding to grain shapes. The nature of the crack is observed at the interface of three or more grain boundaries. In addition, the cracks in samples with different ZrO_2_ content reveal well-developed grain boundaries. It was found that, during sample breaking, there was a fracture at the grain boundary and not through the middle of the grain.

**Figure 7:**
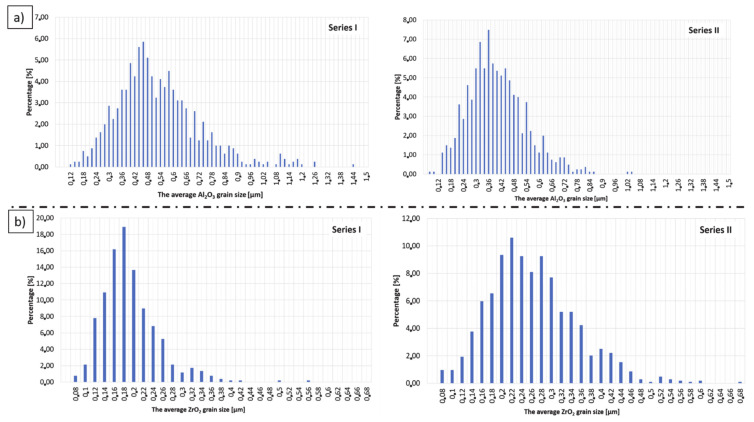
Histograms of medium size Al_2_O_3_ and ZrO_2_ grain distribution in composites from: (**a**) Series I—2.5 vol% ZrO_2_, (**b**) Series II—25 vol% ZrO_2_.

Then, based on the obtained micrographs (Figure 6), a stereological analysis of the studied composites was carried out. Based on the analysis, histograms of medium size Al_2_O_3_ and ZrO_2_ grain distribution were obtained. The histograms are shown in Figure 7. By analysing the results, it is possible to draw the conclusion that the addition of ZrO_2_ effectively limits the growth of Al_2_O_3_ grains. The obtained histograms, regardless of the series, are characterised by a unimodal distribution. Based on the histograms presented in Figure 7a, it was found that in Series I containing 2.5 vol% ZrO_2_, the predominant size of Al_2_O_3_ grains was 0.42–0.46 µm, whereas in Series II containing 25 vol% ZrO_2_, ca. 0.34 µm Al_2_O_3_ grains prevailed. Next, by analysing the histograms presented in Figure 7b, it was observed that in Series I composites, the dominant ZrO_2_ grain size was ca. 0.18, while in Series II it was 0.22 µm.

Table 2 presents the average grain sizes of Al_2_O_3_ and ZrO_2_ in the obtained composites, as determined on the basis of stereological analysis. Based on the results, the mean size of Al_2_O_3_ grains determined for Series I equalled ca. 0.52 µm, while for Series II the mean size of Al_2_O_3_ grains was 0.39 µm. This means that a 10-fold increase in ZrO_2_ content in the composite results in a 25% reduction in the growth of Al_2_O_3_ grains.

The Vickers hardness results revealed the dependence amongst the hardness value and ZrO_2_ phase content of the examined zirconia–alumina composites. It was proven that increasing the amount of ZrO_2_ in the composite results in Vickers hardness values diminishing for the sintered specimens. The average hardness value for the Series I—2.5 vol% ZrO_2_ composites was 18.7 ± 0.74 GPa, while for the Series II—25 vol% ZrO_2_ composites, it was 14.5 ± 0.86 GPa. These results are consistent with the literature data presented by the Pędzich team [35]. His team obtained a hardness of pure ZrO_2_ 3Y-TZ equal to 14.0 GPa for the specimens formed by pressing [35]. Moreover, the research published by Pędzich’s team shows that the samples containing 100 vol% Al_2_O_3_ produced by pressing were characterised by a hardness equal to 17.0 GPa [35]. Therefore, it was found that the addition of 2.5 vol% ZrO_2_ may cause an increase in the hardness value by 0.9% in relation to the materials made of pure Al_2_O_3_. The results reveal that zirconia–alumina composites exhibit a higher hardness value than a material made of pure ZrO_2_. However, it should be emphasised that currently there are no literature reports on zirconia–alumina composites produced by the centrifugal slip casting method. Therefore, it is difficult to directly compare the obtained results with the scientific literature. 

The environmental impacts associated with the raw materials—module A1—and the manufacturing process of zirconia–alumina composite tubes under laboratory conditions—module A3—are presented in Table 3. Based on the obtained results, it can be seen that the extraction of natural resources and the production of the raw materials required for the manufacturing of zirconia–alumina composites cause the emission of greenhouse gases at a level slightly below 2.5 kg CO_2_ eq. per 1 kg of composite materials. This value is comparable with the impact of 1 kg of PVC or PP pipes [44,45] when taking into account that the transportation impact usually constitutes several percentage points of product stage impact (A1–A3) [46,47]. At the same time, the energy resources consumed at modulus A3 for homogenisation, deaeration, drying and sintering are incomparably higher, which can be explained by unit production under laboratory conditions. This result predicts that future optimisation of large-scale processes should include quantitative and qualitative issues related to energy demand. The advantage of ceramic composites over polymer-based solutions can be observed at the use stage of life. Due to the foreseen durability of the zirconia–alumina composite and, hence, the absence of the need for planned or required maintenance operations throughout service life, the environmental impacts associated with the use stage—moduli B1–B7 of LCA—can be deemed as not relevant. What is more, the utilisation of inert ceramic materials eliminates sensitive and commonly ignored environmental issues related to the use of plastic-based materials, such as possible long-term release of microplastics and emissions of harmful substances directly to the soil [48,49].

## 4. Conclusions

Based on the conducted research, the following conclusions were reached:

- The adapted centrifugal slip casting method makes it possible to obtain a zirconia–alumina composite casting in the shape of a sleeve. The obtained composites are characterised by high density equal to 98–99%. It was found that the relative density decreases with increasing ZrO_2_ content in the sinter. The hardness measurement reveals that the Series I—2.5 vol% ZrO_2_ characterised hardness equal to 18.7 ± 0.74 GPa hardness, while for the Series II—25 vol% ZrO_2_ composites, the hardness value was 14.5 ± 0.86 GPa.
- It can be stated that all composites were characterized by a homogeneous microstructure. By analysing the results of stereological analysis, it is possible to draw the conclusion that the addition of ZrO_2_ successfully limits the growth of Al_2_O_3_ grains.
- The life cycle assessment (LCA) of a material manufacturing process developed under the laboratory scale helps to visualise the aspects that have to be considered during the upscaling of the process to industrial size.
- Zirconia–alumina composites constitute an attractive material for construction applications, especially for underground piping systems because they eliminate sensitive and commonly ignored environmental issues related to the use of plastic-based materials, such as possible long-term release of microplastics and emissions of harmful substances directly to the soil.

The trials presented in the manuscript are preliminary studies. The investigations on the mechanical properties of zirconia–alumina composites are in progress and the results will be published in the succeeding papers.

## Figures and Tables

**Table 1:** Summary of density and shrinkage results for zirconia–alumina composites.

Sample	Solid Phase Concentration	Concentration of Phase ZrO_2_	Apparent Density after Sintering	Relative Density	Volumetric Shrinkage	Linear Shrinkage along the Sample
	vol%	vol%	g/cm^3^	%	%	%
Series I	50	2.5	4.0365	99.33	34.19	13.10
Series II		25	4.5009	98.01	35.58	14.40

**Table 2:** Summary of average grain size determined by stereological analysis.

Sample	Average Grain Size [µm]	
	Al_2_O_3_	ZrO_2_
Series I—2.5 vol% ZrO_2_	0.52 ± 0.19	0.16 ± 0.08
Series II—25 vol% ZrO_2_	0.39± 0.13	0.25 ± 0.09

**Table 3:** Environmental characteristics of zirconia–alumina composites containing 2.5% and 25% of ZrO_2_.

Environmental Impacts: (DU) 1 Zirconia–Alumina Tube					
Indicator	Unit	Series I—2.5 vol% ZrO_2_ (51 g)		Series I—25 vol% ZrO_2_ (56 g)	
		A1	A3	A1	A3
Global warming potential	kg CO_2_ eq.	1.16 × 10^−1^	8.04 × 10^0^	1.35 × 10^−1^	8.04 × 10^0^
Depletion potential of stratospheric ozone layer	kg CFC 11 eq.	7.51 × 10^−9^	0.00 × 10^0^	2.21 × 10^−8^	0.00 × 10^0^
Acidification potential of soil and water	kg SO_2_ eq.	8.36 × 10^−4^	1.18 × 10^−2^	8.77 × 10^−4^	1.18 × 10^−2^
Formation potential of tropospheric ozone	kg Ethene eq.	4.71 × 10^−5^	0.00 × 10^0^	4.83 × 10^−5^	0.00 × 10^0^
Eutrophication potential	kg (PO_4_)^3−^ eq.	1.88 × 10^−4^	8.61 × 10^−4^	2.38 × 10^−4^	8.61 × 10^−4^
Abiotic depletion potential (ADP-elements) for non-fossil resources	kg Sb eq.	7.28 × 10^−7^	2.98 × 10^−5^	2.42 × 10^−6^	2.98 × 10^−5^
Abiotic depletion potential (ADP-fossil fuels) for fossil resources	MJ	1.37 × 10^0^	8.03 × 10^1^	1.76 × 10^0^	8.03 × 10^1^
**Environmental Aspects of Resource Use: (DU) 1 Zirconia–Alumina Tube**					
**Indicator**	**Unit**	**Series I—2.5 vol% ZrO_2_ (51 g)**		**Series II—25 vol% ZrO_2_ (56 g)**	
		**A1**	**A3**	**A1**	**A3**
Total use of renewable primary energy resources (primary energy and primary energy resources used as raw materials)	MJ	5.29 × 10^−2^	8.84 × 10^0^	1.73 × 10^−1^	8.84 × 10^0^
Total use of non-renewable primary energy resources (primary energy and primary energy resources used as raw materials)	MJ	1.11 × 10^0^	8.43 × 10^1^	1.41 × 10^0^	8.43 × 10^1^
Use of secondary materials	kg	0.00 × 10^0^	0.00 × 10^0^	0.00 × 10^0^	0.00 × 10^0^
Use of renewable secondary fuels	MJ	0.00 × 10^0^	0.00 × 10^0^	0.00 × 10^0^	0.00 × 10^0^
Use of non-renewable secondary fuels	MJ	0.00 × 10^0^	0.00 × 10^0^	0.00 × 10^0^	0.00 × 10^0^
Net use of fresh water	m^3^	1.48 × 10^−2^	1.00 × 10^−2^	4.50 × 10^−2^	1.00 × 10^−2^
**Other Environmental Information Describing Waste Categories: (DU) 1 Zirconia–Alumina Tube**					
**Indicator**	**Unit**	**Series I—2.5 vol% ZrO_2_ (51 g)**		**Series II—25 vol% ZrO_2_ (56 g)**	
		**A1**	**A3**	**A1**	**A3**
Hazardous waste disposed	kg	7.23 × 10^−7^	0.00 × 10^0^	1.35 × 10^−6^	0.00 × 10^0^
Non-hazardous waste disposed	kg	5.24 × 10^−2^	8.04 × 10^−4^	4.76 × 10^−2^	8.04 × 10^−4^
Radioactive waste disposed	kg	2.21 × 10^−6^	0.00 × 10^0^	3.64 × 10^−6^	0.00 × 10^0^
Components for re-use	kg	0.00 × 10^0^	0.00 × 10^0^	0.00 × 10^0^	0.00 × 10^0^
Materials for recycling	kg	0.00 × 10^0^	0.00 × 10^0^	0.00 × 10^0^	0.00 × 10^0^
Materials for energy recovery	kg	0.00 × 10^0^	0.00 × 10^0^	0.00 × 10^0^	0.00 × 10^0^

## Data Availability

Data sharing not applicable.
